# Improvement of physical and oral function in community‐dwelling older people after a 3‐month long‐term care prevention program including physical exercise, oral health instruction, and nutritional guidance

**DOI:** 10.1002/cre2.226

**Published:** 2019-08-06

**Authors:** Yoshimi Iwao, Hideo Shigeishi, Shouichi Takahashi, Shino Uchida, Shirou Kawano, Masaru Sugiyama

**Affiliations:** ^1^ Department of Public Oral Health, Program of Oral Health Sciences, Graduate School of Biomedical and Health Sciences Hiroshima University Hiroshima Japan; ^2^ Bungotakada City Council of Social Welfare Bungotakada Japan

**Keywords:** older people, physical function, oral function, long‐term care prevention program

## Abstract

**Objective:**

There is limited evidence about the most appropriate way to improve physical and oral function in older people. The aim of this study was to clarify the effectiveness of a long‐term care prevention program combining physical exercise, oral health instruction, and nutritional guidance among community‐dwelling older people.

**Materials and methods:**

We included 43 older people aged at least 65 years (seven men and 23 women; mean age 75.3 years) in Bungotakada City, Japan. The 3‐month program involved a weekly intervention. Physical and oral function was investigated on the first day of the program (i.e., baseline) and at the end of the program (i.e., after 3 months). Physical function was examined using measures such as handgrip strength, timed up‐and‐go test, and one‐leg standing time with eyes open. An oral diadochokinesis test was used to assess oral function.

**Results:**

Mean maximum handgrip strength increased significantly in older people aged ≤74 years (younger participants) and those aged ≥75 years (older participants) after 3 months compared with the baseline. The timed up‐and‐go test duration was significantly lower in older participants after the program. Repetition of the monosyllables *pa* and *ka* and the repetitive saliva swallowing test was significantly improved in both groups. The dysphagia risk assessment for the community‐dwelling elderly score was significantly lower in younger participants after 3 months.

**Conclusion:**

Participation in a 3‐month program combining physical exercise, oral health instruction, and nutritional guidance may contribute to improvement or maintenance of oral and physical function in older people.

## 1 INTRODUCTION

Japan has become a super‐aged society. In 2017, 27.7% of Japan's population was aged ≥65 years, and this proportion is expected to increase to approximately 30% in 2025 (Japan Ministry of Internal Affairs and Communications). To guarantee the comprehensive provision of preventive and healthcare services, nursing care, and life support for older people, the Japanese Ministry of Health, Labor and Welfare has introduced a community‐based integrated care system model (Japan Ministry of Health, Labor and Welfare, 2016). This model allows older people to continue living in their community, even when they require medical or long‐term care. It is also considered important that people can remain physically and mentally healthy, without needing long‐term care, for as long as possible. Participation in social activities can help to maintain health in older people in local communities in Japan (Fukutomi, Kimura, Wada, Okumiya, & Matsubayashi, 2013). Community‐based health programs therefore play a vital role in facilitating healthy aging by maintaining physical and cognitive capacity. Some epidemiological studies have demonstrated that community‐based education and exercise programs can improve physical and oral function in older people (Miyoshi et al., 2019; Ohara et al., 2015; Sakayori et al., 2016; Kaneko et al., 2009). However, there is still limited evidence of the most appropriate way to improve physical and cognitive ability in community‐dwelling older people. The efficacy of combined programs (i.e., those combining physical exercise, psychosocial care, oral health instruction, and nutritional guidance) on the health of older people has also not been fully explored. Recently, the local government of Bungotakada City, Oita Prefecture, Japan, launched a combined program, including physical exercise, oral health instruction, and nutritional guidance, to promote healthy living among older people. It is hypothesized that our health program may improve physical and oral function in community‐dwelling older people. Therefore, we carried out a preliminary prospective cohort study to clarify the effectiveness of this program by investigating changes in physical and oral function in older people before and after the program.

## 2 MATERIALS AND METHODS

### 2.1 Subjects

Since 2015, the local government of Bungotakada City, Oita Prefecture, Japan, has been providing a health program that includes physical exercise, oral health instruction, and nutritional guidance for older people aged ≥65 years living in several local communities. The local government sent invitation letters to older people aged ≥65 years living in three local communities calling for participation in this health program. Fifty‐eight older people participated in this program from May to November 2018. The study design was approved by the Ethical Committee of Hiroshima University (No. E‐2545). To exclude older people with a decline in activities of daily living, older people certified as requiring help (*n* = 0) or long‐term support (*n* = 2), and those with stroke‐related motor paralysis (*n* = 0) were excluded. A further 13 participants dropped out for personal reasons unrelated to the program. The study therefore included 43 older people (seven men, 36 women; mean age 75.3 years) for analysis. All participants signed an informed consent agreement, and almost all 43 participants attended this 3‐month program regularly. The sample size required for the Wilcoxon signed‐rank tests using G*Power (version 3.1.9.4, Heinrich‐Heine‐Universität Düsseldorf, Germany) with a statistical power of 80%, a significance level of 5%, and an effect size of 0.5 was calculated to be 28 subjects. Therefore, the sample size was large enough in this study.

### 2.2 Physical exercise, oral health, and nutritional guidance program

A short‐term 3‐month program with a weekly intervention in community‐dwelling older people was planned for this pilot study. Several studies have investigated the effect of a 3‐month health program on older Japanese adults (Kim et al., 2012; Ohara et al., 2015; Seino et al., 2017; Tamari, Kawamura, Sato, & Harada, 2012). Therefore, we determined that the intervention period of 3 months was appropriate for this study. On the first day of the program, that is, baseline, participants were given a questionnaire about their general and oral health, and their physical and oral function was tested (Table 1). A dental hygienist examined participants' teeth and use of dentures to assess their oral status. The physical exercise (30 min), oral health (40 min), and nutritional guidance (40 min) program started on the second day (Table 1). The physical exercise program involved a stretching exercise, a knee extension exercise, a knee raising exercise, a standing‐on‐one‐leg exercise, and a tiptoe exercise and was developed for community‐dwelling older people (Oita Prefectural Government). This exercise program was supervised by a physical therapist and an occupational therapist. We provided detailed instructions on how to perform the physical exercises effectively via an instruction video. The participants started to exercise according to the instruction video, with assistance from the supervising staff.

**Table 1:** Outline of the long‐term care prevention program combining physical exercise, oral health instruction, and nutritional guidance

	Physical exercise	Oral health program	Nutritional guidance
1st day^a^	−	−	−
2nd day	+	Oral health guidance/tooth brushing instruction with plaque disclosing solution	−
3rd day	+	Lecture on importance of chewing	Introduction to nutrition
4th day	+	Lecture on significant role of saliva/salivary gland massage	Lecture on dehydration prevention and food for dementia prevention
5th day	+	Tooth brushing and denture cleaning instruction	Guidance of cooking practice with nutritionally well‐balanced food
6th day	‐	Oral exercise (i.e., pursed lip exercise, tongue exercise and repeat of syllables/*pa*/, /*ta*/, and/*ka*/)	Cooking practice
7th day	+	Lecture on prevention of aspiration pneumonia	Lecture on healthy food
8th day	+	Lecture on safe swallowing and oral exercise (i.e., holding a straw in the mouth and blowing out through the straw)	Summary of nutrition
9th day	+	Summary of oral health guidance	Guidance of cooking practice with nutritionally well‐balanced food
10th day	−	Oral exercise (i.e., pursed lip exercise, tongue exercise and repeat of syllables/*pa*/, /*ta*/, and/*ka*/)	Cooking practice
11th day^b^	−	−	−
12th day^c^	−	−	−

a Participants were given a questionnaire about their general and oral health, and their physical and oral function was tested.

b Physical and oral function was tested again and the questionnaire was repeated.

c Closing of the program.

The oral health program included oral exercises (e.g., a tongue exercise, a pursed lip exercise, salivary gland massage), tooth brushing instruction, and oral health instruction (e.g., on the role of saliva, the importance of chewing, and prevention of aspiration pneumonia). A dental hygienist provided individual tooth brushing instruction as well as detailed instructions on how to correctly perform the oral exercises.

Nutritional guidance included education on several topics (e.g., healthy food, nutrition and dementia prevention, and dehydration prevention) and cooking practice to prevent malnutrition among older people. This part of the program was provided by a nutritionist. At the end of the 3‐month program, physical and oral function was tested again and the questionnaire was repeated (Table 1).

### 2.3 Physical function test

Physical function was examined using the measures of a handgrip strength test, a timed up‐and‐go test, and a one‐leg standing time with eyes open test. Handgrip strength and one‐leg standing time with eyes open were performed according to the New Physical Fitness Test of the Japanese Ministry of Education, Culture, Sports, Science and Technology (Japan Ministry of Education, Culture, Sports, Science and Technology). The mean peak handgrip strength of both hands was obtained. The participants were asked to raise one leg and stand with their eyes open for a maximum of 60 s. The maximum time for keeping the leg raised was obtained for both legs, and the longer time was used for evaluation. The timed up‐and‐go test measures the overall time it takes individuals to stand up from a chair, walk to a line on the floor 3 m away, turn, return, and sit down on the chair again (Podsiadlo & Richardson, 1991).

### 2.4 Oral function test

To evaluate oral health status, a dental hygienist checked participants' teeth, oral hygiene status, oral mucosal condition, and denture use and performed oral wetness measurement, oral bacterial counts, and oral function tests. An oral diadochokinesis test was used to assess oral motor function (Duffy, 2005). Participants were instructed to pronounce rapid repetitions of single syllables, *pa*, *ta*, and *ka*, as quickly as possible for 5 s. The number of repetitions was automatically measured by a counting device (Kenko‐kun; Takei Scientific Instruments, Niigata, Japan). A repetitive saliva swallowing test (RSST) was used to assess swallowing function. The participants were instructed to swallow saliva as many times as possible over a 30‐s period. Elevation of the thyroid cartilage during swallowing was evaluated by touch. Fewer than three swallows over 30 s was considered a swallowing disorder.

### 2.5 Data collection for oral function

Oral frailty was evaluated using the Kihon Checklist (KCL) developed by the Japanese Ministry of Health, Labor and Welfare. This checklist includes several categories (physical strength, nutritional status, oral function, being housebound, cognitive status, and depression risk; Satake et al., 2016). Oral function was assessed using answers to three questions about difficulty in chewing tough foods, choking or coughing when drinking tea or soup, and feelings of thirst and dry mouth. Based on a previous study, lower oral function was defined as reporting problems in two or more of these questions (Fukutomi et al., 2015).

The risk of dysphagia was evaluated using the dysphagia risk assessment for the community‐dwelling elderly (DRACE; Miura, Kariyasu, Yamasaki, & Arai, 2007). This includes 12 evaluation items (fever, taking a long time to eat, difficulties with swallowing, difficulties with eating hard food, dropping food from the mouth, choking while eating food, choking while drinking liquid, food entering the nasal cavity after swallowing, voice change after eating food, sensation of food stuck in the esophagus, phlegm at the back of the throat, and sensation of food rising into the mouth from the stomach). The answer options were often (score = 2), sometimes (1), and never (0). In line with a previous study, a DRACE score of ≥5 was considered to indicate a high risk of dysphagia (Miura et al., 2007). A DRACE score of <3 was considered a low risk of dysphagia (Miura et al., 2007).

### 2.6 Bacterial count

A bacterial counter (Panasonic Healthcare Co., Ltd., Tokyo, Japan) was used to count the number of oral bacteria. Samples were obtained from the tongue surface using a cotton swab, in line with the manufacturer's instructions. This detection device employs the dielectrophoretic impedance measurement method and ensures that oral bacteria are counted rapidly (Hamada, Suehiro, Nakano, Kikutani, & Konishi, 2011).

## 3 THE WORLD HEALTH ORGANIZATION FIVE WELL‐BEING INDEX

The World Health Organization five well‐being index (WHO‐5) is widely used to evaluate subjective quality of life and psychological well‐being (Bech et al., 2003). We used the simplified Japanese version (S‐WHO‐5‐J; Inagaki et al., 2013). It contains five statements about the last two weeks: “I have felt cheerful and in good spirits”; “I have felt calm and relaxed”; “I have felt active and vigorous”; “I woke up feeling fresh and rested”; and “My daily life has been filled with things that interest me”. It is scored on a three‐point scale from 0 (*at no time*) through 1 (*only a few times*) and 2 (*many times*) to 3 (*all of the time*). The score is calculated by summing the five answers. A higher total score indicates better mental health.

### 3.1 Statistical analysis

Statistical analysis used SPSS software, version 24.0 (IBM Corp., Armonk, NY, USA). The Mann–Whitney U test was used to evaluate significant differences in clinical factors and physical and oral parameters between the groups. Fisher's exact test was used to evaluate significant differences in clinical factors. The Wilcoxon signed‐rank test or McNemar's test was used to assess the differences in physical and oral parameters before and after intervention. Spearman's rank correlation coefficient was used for statistical analysis. *p* values less than.05 were regarded as statistically significant.

## 4 RESULTS

### 4.1 Clinical factors of the study population

The participants' clinical characteristics are summarized in Table 2. The mean age of all participants was 75.3 ± 4.9 years. It was speculated that the degree of physical and oral function improvement may be associated with age in older people (i.e., young‐old or old‐old people). Therefore, we divided participants into two groups (younger participants were people aged ≥65 and ≤ 74 years and older participants were those aged ≥75 years). There was a significant difference in age between the groups. The mean BMI of all participants was 24.9 ± 3.9. The mean number of remaining teeth was 14.0 ± 9.8 in all participants, and 32 of 43 participants (74.4%) used dentures. There were no significant differences in sex, BMI, past medical history, or mean number of remaining teeth between the groups.

**Table 2:** Clinical characteristics of participants

	Participants aged ≥65 and ≤ 74 years (*n* = 17)	Participants aged ≥75 years (*n* = 26)	*p* value
Sex			
Male (7)	2 (28.6%)	5 (71.4%)	.42
Female (36)	15 (41.7%)	21 (58.3%)	
Mean age	70.6 ± 2.6	78.8 ± 3.0	<.001
BMI (kg/m^2^)	24.2 ± 2.7	25.4 ± 4.5	.50
Hypertension			
No (25)	12 (48.0%)	13 (52.0%)	.22
Yes (18)	5 (27.8%)	13 (72.2%)	
Diabetes			
No (37)	14 (37.8%)	23 (62.2%)	.67
Yes (6)	3 (50.0%)	3 (50.0%)	
Stroke			
No (40)	17 (42.5%)	23 (57.5%)	.27
Yes (3)	0 (0%)	3 (100%)	
Heart disease			
No (41)	15 (36.6%)	26 (63.4%)	.15
Yes (2)	2 (100%)	0 (0%)	
Number of remaining teeth	16.4 ± 10.2	12.4 ± 9.4	.22
Denture use			
Non (11)	5 (45.5%)	6 (54.5%)	.73
Denture user (32)	12 (37.5%)	20 (62.5%)	

*Note.* Fisher's exact test was used to evaluate significant differences in clinical factors between the groups. The Mann‐Whitney U test was used to evaluate significant differences in clinical factors such as age, BMI, and number of remaining teeth. *p* < .05 was considered statistically significant.

### 4.2 Comparison of physical function between baseline and 3 months

We examined the change in physical function from baseline to 3 months. At baseline, timed up‐and‐go test duration was significantly longer in older participants than the younger group (*p* = .03). However, there was no significant difference in other physical function measures between the groups at baseline. Mean maximum handgrip strength was significantly higher in both groups at 3 months compared with baseline (Table 3). Timed up‐and‐go test duration was significantly shorter in the older group after the intervention. However, there was no significant difference in timed up‐and‐go test duration in the younger participants. Maximum one‐leg standing time with eyes open was longer after 3 months in both groups, but there was no statistically significant increase.

**Table 3:** Comparison of physical function between baseline and 3 months

	Participants aged ≥65 and ≤ 74 years (*n* = 17)			Participants aged ≥75 years (*n* = 26)		
Physical function measures	Baseline	3 months	*p* value	Baseline	3 months	*p* value
Maximum handgrip strength (kg)	18.6 ± 4.1	20.1 ± 4.9	.003	19.4 ± 8.1	20.7 ± 8.1	.01
Timed up and go test (second)	8.7 ± 1.9	8.3 ± 2.1	.06	10.2 ± 4.5	9.1 ± 2.9	.001
One‐leg standing time with eyes open (second)	35.3 ± 21.1	39.8 ± 19.7	.20	24.6 ± 21.9	24.8 ± 21.4	.82

*Note.* Wilcoxon signed‐rank test for intragroup comparisons were used. *p* < .05 was considered statistically significant.

### 4.3 Comparison of oral function and oral bacterial count between baseline and 3 months

At baseline, there were no significant differences in any oral function measures between the groups. The percentage of participants with KCL score ≥ 2 (i.e., oral frailty) was significantly lower in both groups at 3 months (Table 4). The repetition rate of the monosyllables *pa*, *ta*, and *ka* was significantly higher in younger participants at 3 months. In older participants, there was a significant increase in the repetition rate of *pa* and *ka* but not *ta.* The RSST was fewer than three swallows per 30 seconds in both groups at baseline, indicating that many people in both groups had a swallowing disorder. However, the RSST increased significantly to more than three swallows per 30 seconds in both groups at the end of the program. The mean DRACE score was higher in older than younger participants at baseline and decreased significantly in younger participants at 3 months. The mean DRACE score had also decreased to less than 4 in older participants at 3 months, but this decrease was not significant.

**Table 4:** Comparison of oral function and oral health status between baseline and 3 months

	Participants aged ≥65 and ≤ 74 years (*n* = 17)			Participants aged ≥75 years (*n* = 26)		
Oral function measures	Baseline	3 months	*p* value	Baseline	3 months	*p* value
KCL score (oral function)						
1	10	17	.02	18	24	.03
≥2	7	0		8	2	
Oral diadochokinesis (times/second)						
*pa*	5.9 ± 0.8	6.3 ± 0.9	.02	6.0 ± 0.9	6.4 ± 0.5	.02
*ta*	5.8 ± 0.9	6.2 ± 0.8	.01	5.8 ± 1.0	6.0 ± 0.6	.15
*ka*	5.6 ± 1.0	5.8 ± 0.8	.02	5.5 ± 1.0	5.8 ± 0.7	.001
DRACE score	3.5 ± 2.9	2.1 ± 1.6	.03	4.2 ± 3.6	3.8 ± 3.1	.39
RSST (times/30 seconds)	1.6 ± 1.5	3.6 ± 1.4	<.001	2.1 ± 0.9	3.3 ± 1.3	.001
Oral bacteria number (1.0 × 10^6^ [CFU]/ml)	18.6 ± 14.8	10.9 ± 10.5	.08	14.8 ± 12.8	8.4 ± 7.6	.049

*Note.* Wilcoxon signed‐rank test or McNemar's test for intragroup comparisons were used. *p* < .05 was considered statistically significant.

The oral bacterial count was also lower in both groups at 3 months, with this decrease being significant in older participants.

### 4.4 Comparison of S‐WHO‐5‐J scores between baseline and 3 months

The S‐WHO‐5‐J score was significantly higher in all participants at 3 months (10.5 ± 2.7) compared with baseline (9.4 ± 2.7). The S‐WHO‐5‐J score was also significantly higher in younger participants at 3 months (Table 5). However, there was no significant difference in S‐WHO‐5‐J score between baseline and 3 months in older participants.

**Table 5:** Comparison of S‐WHO‐5‐J scores between baseline and 3 months

	Participants aged ≥65 and ≤ 74 years (*n* = 17)			Participants aged ≥75 years (*n* = 26)		
	Baseline	3 months	*p* value	Baseline	3 months	*p* value
S–WHO–5–J score	9.0 ± 2.6	10.5 ± 2.8	.03	9.7 ± 2.7	10.5 ± 2.7	.09

*Note.* Wilcoxon signed‐rank test for intragroup comparisons were used. *p* < .05 was considered statistically significant.

### 4.5 Comparison of physical function between males and females

There was no significant difference in clinical characteristics such as mean age and BMI among male and female participants (Table 6). However, the percentage of participants with heart disease was significantly higher in males than in females (28.6% vs 0.0%, respectively). Among the physical function tests, maximum handgrip strength was significantly higher in males than in females at baseline. The timed up‐and‐go test was significantly longer in males than in females. Additionally, one‐leg standing time with eyes open was shorter in males than in females, but there was no significant difference. Importantly, timed up‐and‐go test duration decreased significantly in female participants after the intervention (Table 7). However, there was no significant change in physical function test results in males after the intervention.

**Table 6:** Clinical characteristics of male and female participants at baseline

	Male (7)	Female (36)	*p* value
Mean age	75.7 ± 5.6	75.5 ± 4.9	.92
BMI (kg/m^2^)	24.5 ± 2.2	25.0 ± 4.2	.86
Hypertension			
No (25)	4 (16.0%)	21 (84.0%)	1.0
Yes (18)	3 (16.7%)	15 (83.3%)	
Diabetes			
No (37)	6 (16.2%)	31 (83.8%)	1.0
Yes (6)	1 (16.7%)	5 (83.3%)	
Stroke			
No (40)	6 (15.0%)	34 (85.0%)	.42
Yes (3)	1 (33.3%)	2 (66.7%)	
Heart disease			
No (41)	5 (12.2%)	36 (87.8%)	.02
Yes (2)	2 (100%)	0 (0%)	
Number of remaining teeth	14.1 ± 10.6	13.9 ± 9.8	.94
Denture use			
Non (11)	3 (27.3%)	8 (72.7%)	.35
Denture user (32)	4 (12.5%)	28 (87.5%)	
Maximum handgrip strength (kg)	28.9 ± 8.7	17.1 ± 4.3	.001
Timed up and go test (second)	10.8 ± 2.3	9.4 ± 4.0	.02
One‐leg standing time with eyes open (second)	27.6 ± 24.3	29.1 ± 21.9	.83

*Note.* Fisher's exact test was used to evaluate significant differences in clinical factors between the groups. The Mann–Whitney U test was used to evaluate significant differences in clinical factors such as age, BMI, number of remaining teeth, maximum handgrip strength, timed up‐and‐go test, and one‐leg standing time with eyes open. *p* < .05 was considered statistically significant.

**Table 7:** Comparison of physical function between baseline and 3 months in males and females

	Male (7)			Female (36)		
Physical function measures	Baseline	3 months	*p* value	Baseline	3 months	*p* value
Maximum handgrip strength (kg)	28.9 ± 8.7	30.4 ± 9.2	.81	17.1 ± 4.3	18.5 ± 4.4	.21
Timed up and go test (second)	10.8 ± 2.3	10.2 ± 2.4	.54	9.4 ± 4.0	8.5 ± 2.6	.02
One‐leg standing time with eyes open (second)	27.6 ± 24.3	25.4 ± 22.0	.90	29.1 ± 21.9	31.7 ± 21.9	.63

*Note.* The Mann–Whitney U test was used to evaluate significant differences. *p* < .05 was considered statistically significant.

## 5 DISCUSSION

A previous study reported that aging was associated with deteriorated oral function (i.e., decreased oral diadochokinesis and RSST scores) in community‐dwelling older adults aged ≥65 years (Miyoshi et al., 2019). Nearly half of the independent older people in that study were at potential risk of dysphagia when assessed using DRACE scores (Miyoshi et al., 2019). These results suggest that oral frailty is a vital problem in a relatively large number of community‐dwelling older people in Japan. Impaired oral function, including chewing and swallowing disorders, has also been associated with physical frailty and sarcopenia in community‐dwelling older people (Tanaka et al., 2018). Oral rehabilitation as a part of a community‐based social program is therefore expected to improve or maintain oral and physical activity in older people. However, to date, it is not clear which intervention programs aiming to prevent the deterioration of physical function in community‐dwelling older people are likely to be most effective. There are also no standard intervention methods to prevent frailty. It was, however, hypothesized that a multidisciplinary approach (e.g., combining physical exercise, oral health instruction, a dietary approach, psychosocial health care and others) may be appropriate for individual older people and improve their health (Makizako et al., 2015; Serra‐Prat et al., 2017).

We speculated that older participants may exhibit slow or no significant improvement in physical and oral function even after our intervention. In this program, however, physical function, measured using handgrip strength and a timed up‐and‐go test, improved significantly in both younger and older participants after the intervention. This result suggests that the upper limb muscle strength and functional mobility can be improved in independent older people with a 3‐month program. The program also decreased oral frailty and increased the repetition rate of the monosyllables *pa* and *ka* in both groups. However, the repetition rate of *ta* was not significantly increased in older participants. The number of repetitions of the syllable *pa* is related to lip pressure (Tsuga, Maruyama, Yoshikawa, Yoshida, & Akagawa, 2011). Repetition of the syllables *ta* and *ka* has been reported to be associated with lingual motor function (i.e., masticatory function) and tongue pressure (Kikutani et al., 2009; Yamada, Kanazawa, Komagamine, & Minakuchi, 2015). Our results indicate that lip pressure and tongue function may have improved more in younger participants. It is, however, likely that there was a small effect on tongue function in older participants. It may therefore be important to identify oral frailty and try to improve oral function as early as possible.

Analysis of sex‐related differences in physical function revealed that maximum handgrip strength was significantly higher in males than in females at baseline. However, the timed up‐and‐go test was significantly longer in males than in females. Additionally, the timed up‐and‐go test duration decreased significantly in female participants after the 3‐month intervention. Clinical characteristics such as heart disease may have affected mobility in older males. However, it is unclear why only female participants improved their moving capacity after the intervention. Because there was only a small number of older male participants in this study, further research including greater numbers of older male participants is necessary to clarify sex‐associated difference in physical function.

The RSST test revealed that swallowing function improved significantly in both groups after the program. The risk of dysphagia was significantly reduced in younger participants but not in the older group. The mean DRACE score decreased to a level indicating a low risk of dysphagia in younger participants, suggesting that this program might have an effect on the risk of dysphagia in older people when used at a younger age. For more effective improvement in swallowing and oral function in older people, intervention at a younger age should be considered. Additionally, the oral bacterial count was reduced in both groups. This suggests that the oral health program may have improved the participants' oral hygiene status. However, detailed oral health status parameters, such as plaque control indices and periodontal status, were not assessed. Further investigation will therefore be necessary to clarify the association between the oral health intervention and oral hygiene status and periodontal condition.

Subjective quality of life based on positive mood, general interest, and vitality was investigated using the S‐WHO‐5‐J score. The mean S‐WHO‐5‐J score at 3 months had increased to more than 10.0 in both groups. This result indicates that mental health improved after 3 months, as was seen in a previous study using S‐WHO‐5‐J scores (Inagaki et al., 2013).

The intake of an appropriate and balanced diet is a significant factor in healthy aging. However, poor oral health as a result of aging (e.g., from reduced masticatory ability, loss of teeth, poorly fitting dentures, and decreased saliva secretion) worsens the nutritional status of older people (Morisaki, Miura, & Hara, 2015; Walls & Steele, 2004). Maintenance of good oral function therefore plays a significant role in a healthy diet, especially in older people. A combination of oral health and nutritional guidance may be essential in long‐term care prevention programs. In this study, we did not evaluate nutritional status before and after the intervention. Therefore, further studies will be necessary to examine nutritional status to clarify the association between nutritional guidance and improvement in physical and oral function.

Our results suggest that a 3‐month combination program, including physical exercise, oral health instruction, and nutritional guidance, may contribute to healthy aging in older people. However, one limitation of this study was that the overall size of the population was small. Additionally, most participants were healthy and independent older people, who positively participated in this program. There is therefore also a need to investigate the program's efficacy among people who are not actively involved in community health programs.

## 6 CONCLUSIONS

Participation in a long‐term care prevention program may suppress the deterioration of physical and oral function in independent older people. Further studies are needed to clarify the correlation between physical and oral function and the duration of participation in community‐based health programs.

## CONFLICT OF INTEREST

The authors declare that they have no competing interests.
